# Dietary Sugar Shifts Mitochondrial Metabolism and Small RNA Biogenesis in Sperm

**DOI:** 10.1089/ars.2022.0049

**Published:** 2023-05-25

**Authors:** Rashmi Ramesh, Signe Skog, Lovisa Örkenby, Unn Kugelberg, Daniel Nätt, Anita Öst

**Affiliations:** Department of Biomedical and Clinical Sciences, Linköping University, Linköping, Sweden.

**Keywords:** diet, sperm, proteomics, small RNA, mitochondrial ROS, mitochondrial small RNA, miR-10, tsRNA

## Abstract

**Aims:**

Increasing concentrations of dietary sugar results in a linear accumulation of triglycerides in male *Drosophila,* while inducing a U-shaped obesity response in their offspring. Here, using a combination of proteomics and small RNA (sRNA) sequencing, we aimed at understanding the molecular underpinning in sperm for such plasticity.

**Results:**

Proteomic analysis of seminal vesicles revealed that increasing concentrations of dietary sugar resulted in a bell-shaped induction of proteins involved in metabolic/redox regulation. Using stains and *in vivo* redox reporter flies, this pattern could be explained by changes in sperm production of reactive oxygen species (ROS), more exactly mitochondria-derived H_2_O_2_. By quenching ROS with the antioxidant N-acetyl cysteine and performing sRNA-seq on sperm, we found that sperm miRNA is increased in response to ROS. Moreover, we found sperm mitosRNA to be increased in high-sugar diet conditions (independent of ROS). Reanalyzing our previously published data revealed a similar global upregulation of human sperm mitosRNA in response to a high-sugar diet, suggesting evolutionary conserved mechanisms.

**Innovation:**

This work highlights a fast response to dietary sugar in mitochondria-produced H_2_O_2_ in *Drosophila* sperm and identifies redox-sensitive miRNA downstream of this event.

**Conclusions:**

Our data support a model where changes in the sperm mitochondria in response to dietary sugar are the primary event, and changes in redox homoeostasis are secondary to mitochondrial ROS production. These data provide multiple candidates for paternal intergenerational metabolic responses as well as potential biomarkers for human male fertility. *Antioxid. Redox Signal*. 38, 1167–1183.

**Figure:**
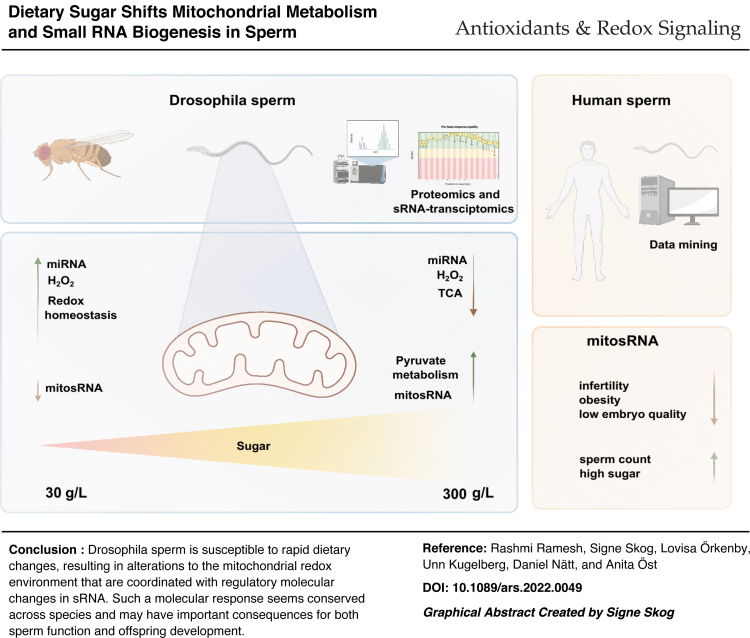


## Introduction

The male germ cell, the harbinger of the subsequent generation's genetic material, is surprisingly sensitive to environmental perturbations. In addition to endogenous factors, environmental and lifestyle-related factors including metabolic disorders such as obesity and type II diabetes impact sperm quality, causing male infertility (Cescon et al, 2020; Chianese et al, 2017; Day et al, 2016; Du Plessis et al, 2015; Katib, 2015; Liu and Ding, 2017; Pergialiotis et al, 2016; Schagdarsurengin and Steger, 2016; Tavares et al, 2016).

Such sensitivity of the sperm has been assumed to be secondary to suboptimal spermatogenesis, and consequently, exploratory studies investigating links between environmental shifts and sperm function have employed long-term or chronic intervention. Recently, however, we and others have found that the sRNA profile in sperm is acutely changed in response to different interventions (Gapp et al, 2021; Nätt et al, 2019; Trigg et al, 2021).

Human sperm change their tsRNA content after just 1 week of high-sugar diet (Nätt et al, 2019); mice exposed to the reproductive toxicant acrylamide change the miRNA content in sperm after 6 days (Trigg et al, 2019); and most dramatic, a few tsRNA are changed in mouse sperm just 3 h after an injection of the corticosteroid dexamethasone (Gapp et al, 2021). A plausible explanation for such rapid responses is that somatic cells in the epididymis deliver material to the sperm *via* exosomes (Sharma et al, 2018; Trigg et al, 2021). Alternatively, since there are reports claiming that the mitochondrial transcription- and translation-machinery is active in mature sperm, the mitochondria could play a central role in the rapid molecular dynamics of the sperm (Gur and Breitbart, 2006; Zhu et al, 2019b).

**Innovation**

This work highlights a fast response to dietary sugar in mitochondria-produced H_2_O_2_ in *Drosophila* sperm and identified redox-sensitive miRNA downstream of this event.

Research in mice has identified sperm sRNA to be the mediator in paternal intergenerational metabolic responses (IGMR) (Chen et al, 2016; Sharma et al, 2016). Thus, the sensitivity of the male germline to environmental changes is sometimes echoed as phenotypic variations in their offspring, suggesting overlapping mechanisms (Nätt and Öst, 2020). In our *Drosophila* model of paternal IGMR, a 2-day diet intervention with different concentrations of dietary sugar results in a linear increase of triglycerides in male founder flies.

In the offspring, however, it promotes a U-shaped obesity response (Öst et al, 2014). The design of the dietary intervention in our human study (Nätt et al, 2019) was motivated by the acute effects we had seen earlier in our *Drosophila* model (Öst et al, 2014). Both dietary interventions were short: the human ∼0.02% of the total lifespan (1 week of 80 × 52 weeks) and the one in *Drosophila* 3.3% (2 days of 60 days). Here, using a proteomic and RNA-sequencing approach, we aimed at expanding our knowledge of rapid molecular changes in sperm in response to diet.

We demonstrate that both proteomic and sRNA profiles in *Drosophila* seminal vesicle and sperm, respectively, are highly dynamic. Since several of the diet-regulated proteins were involved in metabolic/redox regulation, we directly tested whether reactive oxygen species (ROS) would be changed in response to dietary sugar and found a bell-shaped response in sperm mitochondrial ROS production as a function of increasing concentrations of dietary sugar. Such diet-induced ROS production was found to be the driver of diet-induced upregulation of miRNA.

On the contrary, diet-induced upregulation of mitosRNA was not secondary to ROS, thus pointing to a more direct link between diet and sperm mitochondria. Reanalysis of our sRNA-data from human sperm showed that not only mitochondrial tsRNA was changed in response to 1 week of high-sugar diet (as we previously have reported), but also rather there was a global upregulation of sperm mitosRNA.

## Results

### A brief dietary intervention rapidly modifies the sperm proteome

**FIG. 1.**
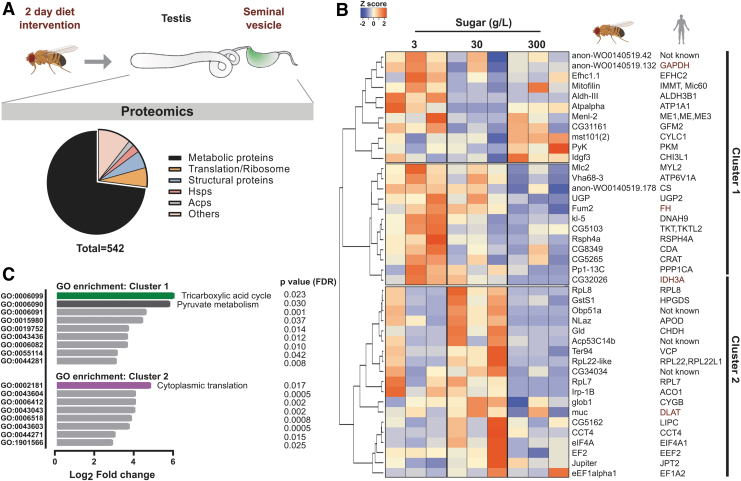
**Rapid response to dietary sugar involves proteomic shifts in sperm. (A)** Schematics of the experimental design and sperm proteome. Two- to three-day-old virgin W^1118^ males were fed a diet containing 3-, 30-, or 300 g/L sugar for 2 days. Seminal vesicles were dissected and subjected to mass spectrometry. Distribution of the proteome is shown in a pie chart. **(B)** Heatmap of significantly changed *Drosophila* sperm proteins (*p* < 0.08) of *n* = 3 experiments with 20 flies per sample, and their human orthologues. Biomarkers of human male infertility is shown in *red*. Hierarchical clustering was performed with Pearson's distance-based complete linkage-based clustering. *Dark bars* on the *right* represents clusters 1 and 2. **(C)** GO term and statistical overrepresentation analyses of proteins in clusters 1 and 2 using pantherDB. FDR, false discovery rate.

We first explored the proteomic landscape of the sperm and sperm microenvironment. For this, seminal vesicles with mature sperm were dissected for protein extraction and subjected to mass spectrometry (Fig. 1A). In total, 542 proteins were identified (Fig. 1A- pie chart and Supplementary Table S1), of which 149 proteins were also found in at least two (out of three) previously published studies of *Drosophila* sperm (Dorus et al, 2006; Takemori and Yamamoto, 2009; Wasbrough et al, 2010) (Supplementary Table S2).

Importantly, the nine most abundant proteins in our dataset were all sperm-specific (Supplementary Fig. S1A), including sperm-exclusive proteins such as Loopin-1 and β-Tubulin 85D (betaTub85D) (Bastian et al, 2021). Functionally, most of the identified proteins were involved in cellular metabolic pathways, whereas others belonged to the broad categories of translation and protein synthesis, heat-shock response, and accessory gland proteins (Fig. 1A- pie chart and Supplementary Table S1). The substantial overlap between our data and previous sperm proteomic studies, along with the high number of sperm-specific proteins, justified further functional investigation in relation to dietary intervention.

Three groups of adult virgin male flies were, therefore, fed differing amounts of sugar (10-fold increases viz 3, 30 or 300 g/L) for 2 days (Fig. 1A). Sperm-specific proteins, such as Loopin-1 and betaTub85D, remained stable in all three dietary conditions (Supplementary Fig. S1A), whereas a subset of proteins were identified as differentially expressed (Fig. 1B). Unsupervised hierarchical clustering of the differentially expressed proteins (*p* < 0.08) revealed two major clusters (Fig. 1B, clusters 1 & 2).

Proteins involved in TCA cycle (CS, Fum2, CG32026, Irp-1B) and pyruvate metabolism (PyK, Gapdh, and Menl-2) were enriched in cluster 1, whereas translation-related proteins dominated cluster 2 (RpL22-like, RpL7, RpL8, eIF4A, eEF2) (Fig. 1C).

To gain more translational and functional insights, we studied their human orthologues *in silico*. Interestingly, many of the proteins present in Figure 1B had previously been reported in human male infertility (CTD Gene-Disease Associations dataset) (Rouillard et al, 2016). Among these were well-studied protein biomarkers for male infertility such as IDH3A, GAPDHS, FH, and DLAT (Fig. 1B, red text) (Agarwal et al, 2020; Torra-Massana et al, 2021). This translational overlap supports the hypothesis of a link between paternal diet and infertility.

In fact, germline knockdown of *eIf4a*, *cct4* and *mitofilin* (Supplementary Fig. S1B), resulted in a strong infertility phenotype in flies, thus highlighting the role of such proteins in spermatogenesis (representative micrographs for eIF4A knockdown in Supplementary Fig. S1C).

In summary, our findings highlight the rapid shift in the sperm proteome related to metabolism and translation after a brief dietary intervention. Notably, some human orthologues of the shifted proteome are implicated in male infertility, providing important clues about a mechanism associating diet with male infertility.

### Diet acutely modulates ROS production in the sperm

**FIG. 2.**
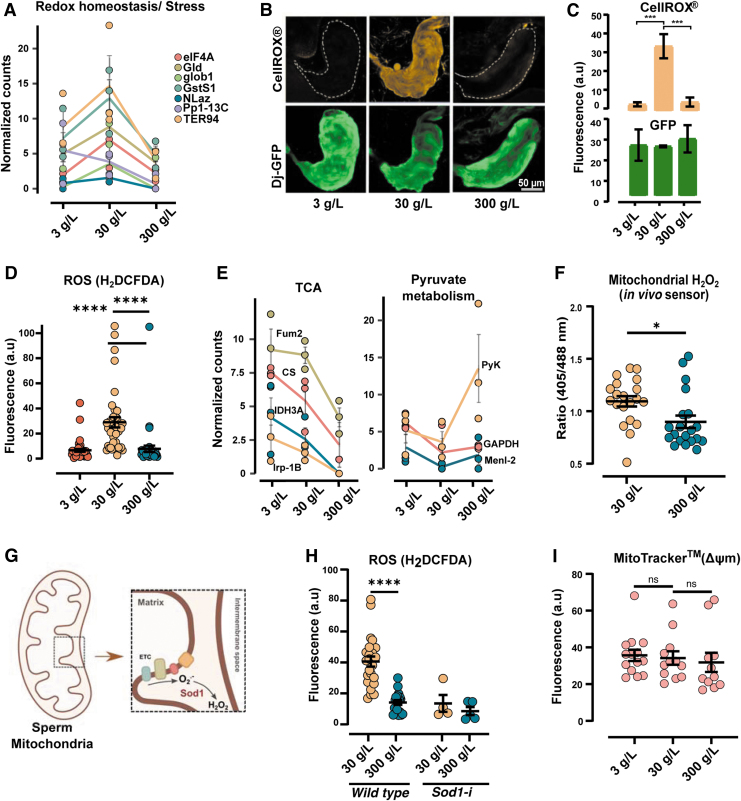
**Paternal diet rapidly changes mitochondrial ROS production in sperm. (A)** Proteins involved in stress/redox homeostasis from Fig. 2B. Each individual sample is presented as *closed circles*. Matching colored lines is plotted as the mean level of each protein of *n* = 3 experiments ± SEM. **(B)** Representative images of seminal vesicle with ROS in mature sperm in *orange* channel (CellRox^®^ *orange*) and Dj-GFP (sperm tail) in *green* channel. Scale bar = 50 μm. **(C)** Seminal vesicles as in **(B)** were quantified using Fiji. Mean intensity of CellRox *orange* is increased in the 30 g/L sugar condition (***p ≤ 0.001) of *n* = 4–6. **(D)** Quantification of seminal vesicle labeled with H_2_DCFDA. Mean intensity is increased in the 30 g/L sugar condition, (****p ≤ 0.0001) of *n* = 38–45. **(E)** Proteins involved in TCA and pyruvate metabolism from Figure 2B were plotted separately to indicate expression trends across the three diets. Each protein is presented as a colored *closed circle*. Matching colored lines are plotted as the mean level of each protein of *n* = 3 experiments ± SEM. **(F)** Quantification of redox changes in mitochondrial H_2_O_2_ in seminal vesicles of flies expressing the mito-roGFP2-Orp1 ratiometric sensor. Ratio of fluorescence between 405 and 488 nm was plotted on *y* axis, and diets on *x* axis. *n* = 20, (*p ≤ 0.05). **(G)** Cartoon representation of sperm mitochondria, with the ETC zoomed in (*dotted box*). Complexes I, II, III, and IV of the ETC are highlighted, and superoxide production from oxygen is indicated (O_2_^.−^) in the intermembrane space. Sod1 in the mitochondrial intermembrane space catalyzes the conversion of O_2_^.−^ to H_2_O_2_. **(H)** Quantification of ROS in wild-type flies and in flies with germline specific knockdown *via* RNAi of Sod1 (Sod1-i), with indicated diets. *n* = 4–5 (Sod1-i) and *n* = 18–24 (wild type). **(I)** Quantification of ΔΨm. Fluorescence intensities from seminal vesicles stained with Mitotracker™ CMXRos. *n* = 11–14. All data shown are mean ± SEM. Samples are compared using unpaired *t*-test. a.u = arbitrary units, ns = not significant. ETC, electron transport chain; ΔΨm, mitochondrial potential; TCA, tricarboxylic acid cycle.

Careful inspection of the differentially expressed proteins (Fig. 1B) revealed a subset of proteins that are involved in stress/redox homeostasis (Fig. 2A). The expression of these proteins, namely GstS1, Nlaz, Gld, eIF4A, TER94, glob1, and Pp1–13C, was higher in 30 g/L compared with the other two diets (Fig. 2A). We, therefore, hypothesized that the flies eating 30 g/L sugar would differ in sperm ROS production compared with the flies eating 3 or 300 g/L sugar.

Seminal vesicles from flies with a sperm-specific GFP-reporter, *Don juan* GFP (*Dj*-GFP), that ate diets containing 3-, 30-, or 300 g/L sugar for 2 days, were dissected and incubated with an ROS-indicator (CellROX^®^ Orange). Visualization of the live tissues revealed a striking difference in ROS production in response to diet (Fig. 2B). Sperm from 30 g/L sugar diet showed distinct orange fluorescence indicative of active ROS production, whereas negligible to no fluorescence was detected in low- and high-sugar diets (Fig. 2B, C, upper panels).

The differences in fluorescence were not a result of changes in the amount of sperm *per se*, since GFP fluorescence corresponding to *Dj*-GFP from sperm tails remained unchanged (Fig. 2B, C, lower panels). We also independently replicated the findings in a separate fly strain (W^*1118*^) using a general ROS indicator dye (H_2_DCFDA) (Fig. 2D). Together, this clearly suggests that a diet comprising 30 g/L sugar promotes ROS production in the sperm of *Drosophila melanogaster*.

It has been reported that sperm mitochondria are major sources of ROS (Koppers et al, 2008; Kothari et al, 2010), and since we found proteins involved in TCA cycle to be upregulated in seminal vesicles of 30 g/L sugar-eating males, whereas proteins involved in glycolysis were downregulated (Figs. 1B and 2E), we hypothesized that diet-induced ROS originated from the mitochondria. We, therefore, used a previously characterized ratiometric H_2_O_2_ redox sensor, the mitochondrial-roGFP2-Orp1 (Albrecht et al, 2011) (Supplementary Fig. S2A–C).

This *in vivo* sensor allowed us to specifically track mitochondrial ROS, with the added advantage of being a selective ROS-reporter for H_2_O_2_. As previously described (Fig. 1A), 3–5-day old male flies were fed 30- or 300 g/L sugar for 2 days, and fluorescence was measured using confocal microscopy by sequential excitation at 405- and 488 nm (See methods section for details). In line with the TCA-related protein changes, we found that the 30 g/L sugar condition resulted in seminal vesicles with a higher 405/488 ratio, thus supporting a diet-sensitive mitochondria-driven H_2_O_2_ production in sperm (Fig. 2F and Supplementary Fig. S2C).

It is known that superoxide radicals produced from the electron transport chain (ETC) are converted to H_2_O_2_ by the enzyme superoxide dismutase 1 (Sod1) (Fig. 2G). We knocked down mitochondrial Sod1 (denoted as Sod1-i) in the germline by RNA interference and measured ROS levels by microscopy. As expected, quantification of fluorescence in seminal vesicles from Sod1 knockdowns revealed that inhibiting the sperm's ability to convert superoxide radicals to H_2_O_2,_ attenuated the diet-induced ROS production (Fig. 2H).

We next turned to investigating whether mitochondrial function itself was modulated by diet. Since 30 g/L sugar favored ROS production in comparison to 3- and 300 g/L, we reasoned that mitochondrial activity could be altered. We, therefore, measured mitochondrial potential (ΔΨm). Seminal vesicles from flies eating 3-, 30-, or 300 g/L sugar for 2 days were dissected and incubated with a fluorescent dye that specifically stains the mitochondria (MitoTracker™ Red CMRos). The uptake of this cell-permeable dye is dependent on ΔΨm, whereby a strong signal of the dye indicates a high ΔΨm.

Bright red fluorescence was seen in seminal vesicles from flies feeding 3-, 30-, or 300 g/L sugar (Supplementary Fig. S2D). Quantification of red fluorescence in seminal vesicles (containing mature sperm) revealed no significant differences in sperm of either diet (Fig. 2I). These results suggest that although diet had a rapid effect on ROS production in sperm, there was little to no effect on the mitochondrial ΔΨm in mature sperm. Although a general change in ΔΨm was not detected, it is possible that specific sites in the ETC are affected by diet.

We also measured ATP generation as another parameter to determine mitochondrial function in sperm and found that the total ATP production in pure sperm did not differ with diet (Supplementary Fig. S2E). Thus, future studies on dissecting the role of individual complexes are needed. The rapid changes in the sperm mitochondrial H_2_O_2_ production after a diet of just 2 days, are, however, very clear.

### Acute dietary intervention rapidly modifies the sRNA profiles in sperm

**FIG. 3.**
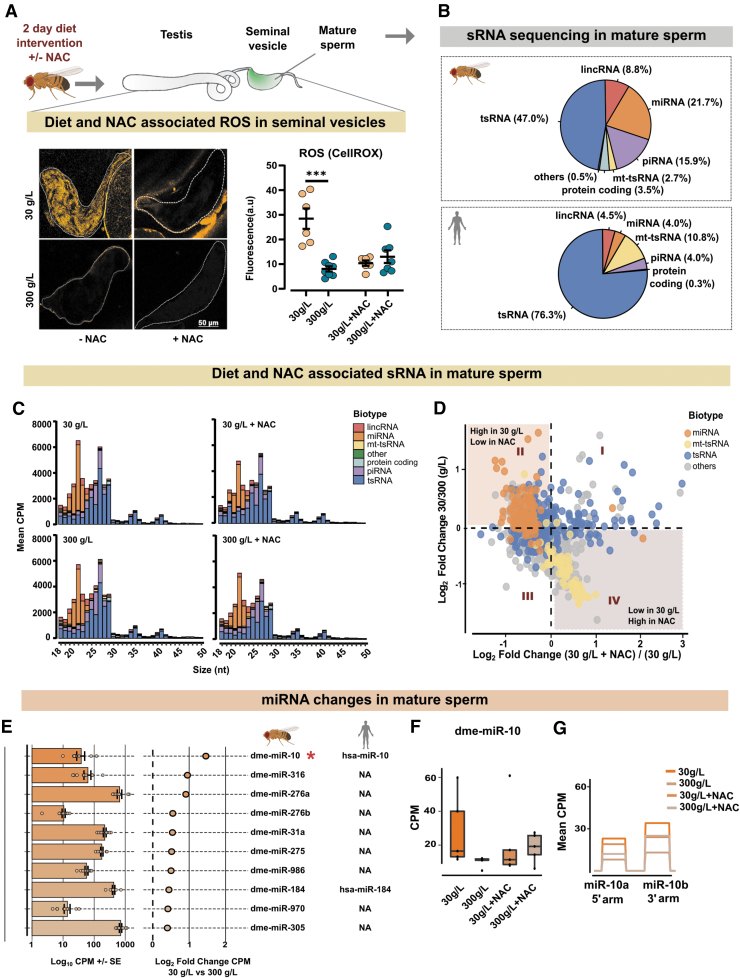
**Rapid alteration in sperm sRNA following dietary intervention. (A)** Following a 2-day dietary intervention with or without NAC, seminal vesicles were dissected, and ROS levels were visualized and quantified as earlier. The 30 g/L sugar diet increased ROS (***p ≤ 0.001, unpaired *t*-test) of *n* = 6–8, whereas addition of NAC mitigated this. Results are mean ± SEM. On a separate set of flies from the same experiment, sperm was isolated and sequenced for sRNA. **(B)** Comparison of sRNA in fly and human sperm. Human sperm sRNA data were derived from (Nätt et al, 2019). Pie charts are percent CPM of perfect matches to respective reference genome. rsRNA are not shown. snoRNA (less than 2%), snRNA (less than 1%), and reads with no annotation (less than 1%) are classified as “others.” Fly data *n* = 20, human data *n* = 15. **(C)** Size distribution of sRNA in Drosophila sperm in the diets with and without NAC. Reads mapping to rsRNA are excluded. Reads mapping to snoRNA, snRNA, and those sRNA that map to the reference genome but not to any other sRNA-subtypes are grouped as “other.” **(D)** Scatter plot of fold changes showing the distribution of sRNA in the tested conditions. *x* axis represents Log2-fold change (30 g/L+NAC)/(30 g/L), and y axis represents Log_2_-fold change (30 g/L)/(300 g/L). Each *closed circle* represents individual sequence, in total 983. miRNA (*orange*), mitochondrial tRNA (*yellow*), tRNA (*blue*), and others (*gray*) are shown. Reads mapping to rsRNA are not included. **(E)** miRNA identified in this study. Fly miRNA are indicated under the fly pictogram. Bars represent mean CPM. The corresponding *closed circles* represent mean Log_2_-fold change. Error bars are ± SE, *p ≤ 0.05, Negative Binomial Generalized Linear Model. Human orthologues are indicated under the human pictogram. **(F)** Bar graph for CPM expression of dme-miR-10 in all four dietary groups. Each point represents the mean value of the two miRNA sequences mapping to dme-miR-10 for each sample. **(G)** Coverage plot of dme-miR-10 showing expression of both the 5′ (dme-miR-10a) and 3′ arms (dme-miR-10b). Each colored line represents the mean CPM of indicated dietary condition. CPM, counts per million; NA, not available; NAC, N-acetyl cysteine; ROS, reactive oxygen species.

Having found that mitochondrial ROS respond rapidly to diet, we sought to explore whether sperm sRNA profiles were similarly shifted, and if so, whether such changes would be secondary to diet-induced ROS. For this, we took advantage of the direct quenching of ROS with the general antioxidant N-acetyl cysteine (NAC) supplemented in diet containing either 30- or 300 g/L sugar (Fig. 3A). After 2 days on the diet, the seminal vesicles were dissected and incubated with CellROX, and ROS was measured by microscopy and quantified (Fig. 3A).

As previously observed, the 30 g/L sugar diet resulted in high ROS production in sperm, whereas flies fed 300 g/L sugar had little to no ROS (Fig. 3A, −NAC). The NAC supplementation (1 mg/mL) in both 30- and 300 g/L diets greatly diminished the ROS levels (Fig. 3A, +NAC), proving the effectiveness of the antioxidant in quenching the diet-induced ROS in seminal vesicles. We, therefore, used NAC to investigate the effect of diet-induced ROS on sperm sRNA. Importantly, to eliminate the contribution from somatic sRNA, only pure mature sperm was used for sRNA sequencing (Fig. 3A and Supplementary Fig. S3A, lower panels).

Sperm sRNA sequencing revealed the presence of different sRNA biotypes, including, in order of their abundance: rRNA-derived small RNA (rsRNA), tRNA-derived small RNA (tsRNA), microRNA (miRNA), piwi-interacting RNAs (piRNA), long intergenic non-coding RNA (lincRNA), sRNA from protein-coding RNA, and mitochondrial tRNA-derived small RNA (mt-tsRNA) (Supplementary Table S3). For comparison, we analyzed human sperm sRNA data from Nätt et al (2019).

Reads mapping to rsRNA were dominant in the datasets, from both flies and humans (94% *vs.* 74% respectively, see Supplementary Table S3 for rsRNA information in fly sperm). We did not detect major changes in the nuclear rsRNA profiles in response to diets and therefore excluded them from further analyses.

A side-by-side comparison of sRNA from flies and human sperm (Nätt et al, 2019) revealed similarities in the distribution and abundance of various sRNA biotypes, with tsRNA being the most abundant biotype in the sperm of both species (Fig. 3B). Next, we looked at the size distribution of various sRNA biotypes in the sperm of flies fed with 30- and 300 g/L sugar, with and without NAC (Fig. 3C). The size distribution showed diverse lengths of tsRNA spanning 18–50 nucleotides, and a clear peak of miRNA at 21–23 nt (Fig. 3C).

On a gross level, we could not see any changes of tsRNA, but we noticed that the ROS-inducing diet was associated with a larger peak of miRNA. Further analysis confirmed this and revealed a general upregulation of miRNA in response to diet (Fig. 3D, E, Supplementary Fig. S3B and Supplementary Table S4). As indicated by the miRNA peaks of the size distribution graphs (Fig. 3C), supplementing food with NAC attenuated the upregulation of miRNA in the ROS-inducing diet (Fig. 3D and Supplementary Table S4).

Despite the general upregulation of miRNA, on an individual level only one miRNA, dme-miR-10, was found to be significantly changed in the 30 g/L condition as compared with the 300 g/L diet condition (*p* < 0.05, Fig. 3E asterisk *, F). Both the 5′ and 3′ arms of miR-10, called miR-10a and miR-10b respectively, were upregulated in the 30 g/L sperm and similarly quenched by NAC (Fig. 3G). Next, we performed additional sequencing of sperm from flies fed 3, 30, or 300 g/L and analyzed these in isolation (Supplementary Table S6). A comparison of all three diets revealed a bell-shaped response, with reduced expression of miR-10 in sperm from flies fed the 3- or 300 g/L sugar diet compared with 30 g/L (Supplementary Fig. S4A, B and Supplementary Table S7).

### *Drosophila* sperm mitosRNA is increased in response to a high-sugar diet

**FIG. 4.**
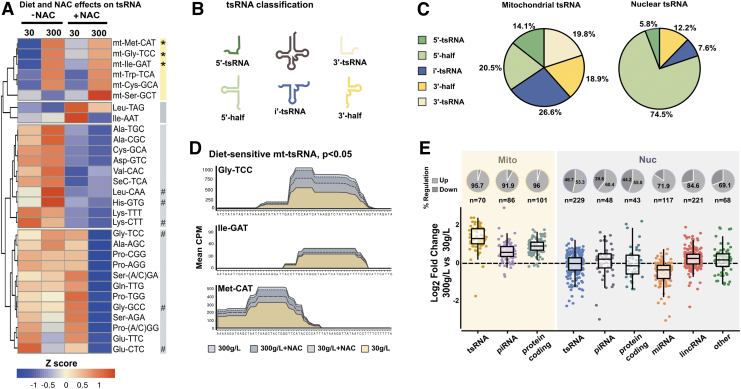
**Mitochondrial sRNA are increased in fly sperm after a short-term high sugar diet. (A)** Heatmap showing expression of tsRNA. TsRNAs that originate from the same mature tRNA are combined. Each *column* represents the mean CPM expression from the indicated dietary condition. Clusters are based on unsupervised clustering and are numbered 1–4 in *red*. The “mt” in cluster 1 indicates tsRNA of mitochondrial origin. *p ≤ 0.05, as determined by Negative Binomial Generalized Linear Model. Hashtag (#) refers to tsRNA identified in other studies (Chen et al, 2016 and Sharma et al, 2016). **(B)** Illustrative representations of the fragments derived from tRNA analyzed in this study. See the Methods section for details on tsRNA classification. **(C)** Distribution of tsRNA based on their mapping to nuclear and mitochondrial genomes. For each tsRNA, the percentage of mean CPM is presented. **(D)** Coverage plots of significantly altered mitochondrial tsRNAs, namely Gly-TCC, Ile-GAT, and Met-CAT. *x* axis shows the corresponding mature tRNA sequence, and y axis represents the mean CPM values. **(E)** Fold changes 300- *versus* 30 g/L of the indicated sRNA biotypes, where biotypes are separated independent of genomic origin. Transcripts from mitochondrial genome are presented to the *left* in *yellow box*, and transcripts from the nuclear genome are presented to the *right* in a *blue box*. Pie charts above each bar represent the percentage of upregulated and downregulated transcripts, respectively. Each *dot* represents a unique transcript, and *n* values per group are presented in the graph.

We have previously found that 1 week of high-sugar diet in humans changed the amount of nuclear internal tRNA fragments (nitRNA), as well as most mt-tsRNA in sperm. We, therefore, analyzed the tsRNAs in both 30- and 300 g/L sugar diets with and without NAC (Supplementary Table S5). In concordance with our previous findings in human sperm, unsupervised hierarchical clustering of tsRNAs separated the nuclear and mitochondrial tsRNA to distinct clusters (Fig. 4A). Three mt-tsRNAs were found to be significantly upregulated with the high-sugar diet (Fig. 4A, asterisks*).

These three mt-tsRNA, Ile-GAT, Gly-TCC, and Met-CAT, were upregulated in sperm from the high-sugar diet eating males regardless of the addition of NAC to the food (Fig. 4A, D). Thus, diet-sensitive mt-tsRNA are produced in a pathway independent of diet-induced mitochondrial ROS.

We then analyzed transcripts based on cut-sites using the bioinformatic package Seqpac (Skog et al, 2021) and with information about tRNA loop structure taken from tRNAscan-SE (Lowe and Chan, 2016). Five sub-types of tsRNA were defined: 5′-half, 5′-tsRNA, i′-tsRNA, 3′-tsRNA, and 3′-half (Fig. 4B). Studying the genomic source of these transcripts, the distributions of the tsRNA subtypes varied depending on whether they were of nuclear or mitochondrial origin (Fig. 4C).

The nuclear tsRNA were dominated by 5′-halves (Fig. 4C-nuclear tsRNA), as previously reported (Bui et al, 2018; Koppers et al, 2008; Nätt et al, 2019), whereas the subgroup most present in mt-tsRNA was i′-tsRNA fragments (Fig. 4C-mitochondrial tsRNA). The mt-tsRNA that were significantly changed by diet are mainly i′-tsRNA fragments (Fig. 4D). We next extended our analysis to investigate the changes in all sRNA separated on genomic origin and found a global upregulation of mitosRNA in the sperm of high-sugar diet eating flies (Fig. 4E).

Interestingly, mt-tsRNA were found to be decreased in 3 g/L compared with the 30 g/L condition in all but three mt-tsRNA, suggesting a linear response to dietary sugar levels in those mt-tsRNA (Supplementary Fig. S5A, B and Supplementary Table S8).

### Human sperm mitochondrial sRNA are dynamic and correlate with sperm quality

The finding that a short intervention with a high-sugar diet resulted in an increase of mitosRNA in *Drosophila* (Fig. 4E) prompted us to investigate whether a short-term high-sugar diet in humans generates a similar response in sperm. We have earlier performed a human study where we recruited young healthy males, measured their weight, height, and activity level so that we could tailor meals based on their individual recommended daily intake (RDI) (Nätt et al, 2019).

**FIG. 5.**
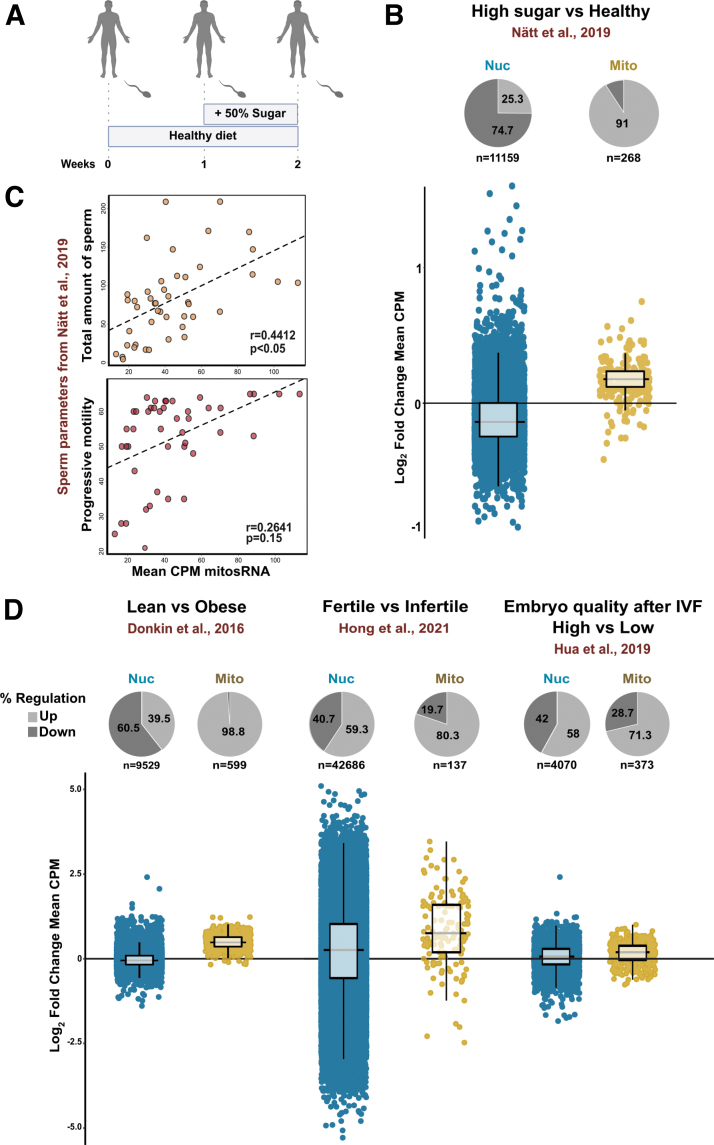
**Human sperm mitochondrial sRNA are upregulated in high-sugar diet, and they are positively correlated with sperm quality. (A)** Study design of Nätt et al (2019). Fifteen healthy male participants were given a diet based on Nordic nutritional recommendations for 1 week. The following week, an additional 50% of the recommended daily intake of sugar was added. Sperm samples were collected at three time points, beginning of week 0, end of week 0, and end of week 1. **(B)** Log_2_-fold change of global expression levels of small RNA transcripts mapping perfectly to either the nuclear or the mitochondrial human genome in data from **(A)**. Every *dot* represents the Log_2_-fold change mean of one transcript. **(C)** Repeated-measure correlation of mean mitochondrial sRNA levels per individual and sample session total amount of sperm (*top panel*) and progressive motility (*bottom panel*). r = reported repeated-measure correlation coefficient. *p* = reported *p*-value for repeated-measure correlation coefficient. Repeated-measure correlation analysis was performed using rmcorr ver. 0.45. **(D)** Log_2_-fold change of global expression levels of small RNA transcripts in published human sperm sRNA datasets as indicated.

Based on their RDI, participants first received a healthy diet based on Nordic nutrition recommendations (Norden.org, 2012), for 1 week to establish a baseline (Fig. 5A). This week was directly followed by 1 week with a high-sugar diet. Sperm samples were collected both before and after a high-sugar intervention and subjected to sRNA-seq.

Re-analyzing this dataset showed that 1 week of high-sugar diet, indeed, resulted in a mean increase of mitosRNA in human sperm (Fig. 5B). Other physiological data and semen parameters were also available from Nätt et al (2019). Using these, we found a significant positive correlation between mitosRNA and total amount of sperm (Fig. 5C top panel) and progressive sperm motility (Fig. 5C bottom panel). These findings implicate mitosRNA as indicators of sperm quality.

To the best of our knowledge, mitosRNA have not been extensively analyzed in connection with sperm quality. Therefore, we analyzed mitosRNA from several human studies. In doing so, we found that data from Donkin et al (2016) revealed a higher expression of mitosRNA compared with obese individuals (Fig. 5D). To strengthen the link between mitosRNA and sperm quality, we also analyzed the sRNA data from fertile controls and patients with asthenozoospermia from Hong et al, (2021), and we found higher levels of mitosRNA in the sperm of fertile men (Fig. 5D).

Finally, to get an insight of whether sperm mitosRNA is relevant for embryonic development, we analyzed mitosRNA from data generated by Hua et al (2019). Remarkably, sperm that generated high-quality embryo at IVF according to Hua et al (2019) also had higher levels of mitosRNA (Fig. 5D).

## Discussion

Here, we show that *Drosophila* sperm are acutely sensitive to dietary sugar. Although the bulk of structural proteins were unaffected by diet (Supplementary Fig. S1B), we found changes in proteins involved in metabolism and redox homeostasis (Figs. 1A, B and 2A). This change in redox homeostasis was reflected in ROS production in sperm mitochondria, which dramatically increased with 30 g/L sugar (Fig. 2B–D, F and Supplementary Fig. S2C). In parallel, high-throughput sequencing of purified sperm RNA revealed coordinated changes in the sperm sRNA. In-depth analyses of the sRNA profiles indicated that response to diet in sperm manifested in both ROS-dependent and -independent ways.

The NAC-mediated depletion of ROS in sperm was able to reverse the expression of diet-altered miRNA, in particular miR-10 (Fig. 3D, E), suggesting this to be secondary to ROS. Moreover, we found that high-sugar diet suppressed the production of ROS, while increasing the expression of the mitosRNAs (Fig. 4A, D, E). The mitosRNA were also increased in the human sperm after a short high-sugar diet intervention (Fig. 5B), suggestive of evolutionary conserved mechanisms where mitochondrial metabolic flexibility and mitosRNA biogenesis are in the center of diet-dependent molecular responses in the sperm.

The existence of a gut-gonad axis has recently been demonstrated in *Drosophila*, wherein the male intestine secretes citrate to the adjacent testes and promotes sperm maturation (Hudry et al, 2019). A similar gut-gonad axis was recently described in a sheep model of diet-induced metabolic syndromes (Zhang et al, 2022). In both these models, metabolic perturbations altered spermatogenesis and sperm numbers. However, with our short dietary intervention, in flies and humans (Nätt et al, 2019), the total amount of sperm was not affected by diet.

From experiments in flies, two findings indicate unchanged sperm numbers: (1) highly expressed sperm proteins such as loopin-1 and β-tubulin are maintained at the same level in the proteomics data independent of diet (Supplementary Fig. S1A), and (2) microscopy of seminal vesicles of the sperm-specific fusion protein Dj-GFP revealed no change in fluorescence intensity (Fig. 2C). In addition, staining of seminal vesicles with MitoTracker Red CMXRos showed similar staining patterns across the tested diets (Fig. 2I and Supplementary Fig. S2D), revealing negligible to no changes in sperm numbers or in mitochondria potential.

These findings demonstrate a metabolic plasticity in sperm following a short-term dietary change, rather than changes to the sperm numbers *per se*. It is, however, possible that long-term dietary changes, or other nutrient compositions, would impede spermatogenesis and modulate the number of sperm being produced.

Although sperm numbers appeared unchanged by diet in flies, we observed that diet altered the mitochondrial H_2_O_2_ production in sperm. Most studies linking glucose and sperm ROS have been carried out *in vitro*, and there is yet no consensus on dose-dependent response of glucose on ROS production in the sperm. In human sperm, for example, glucose has been shown to not affect ROS levels (Portela et al, 2015). However, under capacitation conditions, basal level of ROS is produced by sperm, irrespective of the presence or absence of glucose (Carrageta et al, 2020).

On the contrary, boar sperm show an inverse relationship between mitochondria-produced ROS and glucose levels (Zhu et al, 2019a). *In vivo*, a low-protein diet has been shown to increase ROS in mice spermatozoa (Yoshida et al, 2020), but, to the best of our knowledge, there are no studies linking dietary sugar intake to sperm ROS. There are a few interesting studies highlighting the plasticity of human sperm.

For example, the human spermatozoal epigenome is dynamically remodeled after bariatric surgery (Donkin et al, 2016). Moreover, a short-term endurance training changes the sRNA in the sperm (Ingerslev et al, 2018). It is, however, unknown, whether this plasticity relates to metabolic shifts in sperm mitochondria.

The mechanisms linking dietary sugar to sperm mitochondrial H_2_O_2_ production also remain unclear. The most straightforward explanation is that the mitochondrial H_2_O_2_ production correlates with the amount of ATP being produced *via* oxidative phosphorylation in the ETC. It can be envisioned that the 30 g/L diet, being close to a caloric restricted diet, puts the fly in a starvation mode and forces it to use its triglyceride stores to maintain ATP production *via* beta-oxidation and oxidative phosphorylation, whereas the high-sugar diet allows for some ATP to be produced *via* glycolysis.

The 3 g/L is a very low-caloric diet that will lead to death by starvation in approximately a week, so it is possible that the fly's metabolism in this condition is decreased to a minimum, thereby producing low amounts of mitochondrial H_2_O_2_. Such metabolic flexibility is in line with reports claiming that sperm can utilize diverse metabolic strategies and that they respond to changes in nutrient composition of their media *in vitro* (Ortiz-Rodríguez et al, 2021; Peña et al, 2022).

Given that ROS is required for sperm functions in humans (Du Plessis et al, 2015; Dutta et al, 2020), the modulation of such essential processes by diet is intriguing and can have far-reaching consequences. Given its easy diffusibility across membranes, and a longer half-life, H_2_O_2_ is considered one of the main signalling molecules among the ROS species (Holmström and Finkel, 2014; Rhee, 2006; Veal et al, 2007). On the other hand, ROS-induced oxidative stress can lead to male infertility (Agarwal et al, 2019; Agarwal et al, 2008; Barati et al, 2020; Bui et al, 2018; Tremellen, 2008). Therefore, the production of ROS must be counterbalanced by antioxidant systems. In mammals, it is well known that proteins secreted from the epididymis provide such protections for the maturing sperm [reviewed in (Chianese and Pierantoni, 2021)].

Since tsRNA halves have been shown to be produced in response to ROS (Thompson et al, 2008) and have been suggested by us and others to be the driver of tsRNA generation in sperm (Nätt and Öst, 2020; Zhang and Chen, 2020), we investigated this in detail here. Although we cannot find a clear diet-ROS-tsRNA axis, we do find major shifts in the tsRNA groups in response to the antioxidant NAC (Fig. 4A). Most interestingly, when supplemented with NAC there is a dietary effect on several of the classical stress-induced tsRNA such as Gly-TTC, Gly-GCC, and Glu-CTC.

Clearly, more research is needed to understand the complex relationship between diet, ROS, and tsRNA in sperm. In contrast to nuclear tsRNA, mitochondrial-derived tsRNA show a clear dependence of diet and independence of NAC. Not much is known about mitochondrial-derived sRNA, but they have been detected in multiple tissues including ovaries, testis, and cancer cell lines (Kiani et al, 2019; Salas-Huetos et al, 2020). Importantly, mitosRNA have been proposed to regulate mitochondrial gene expression, possibly acting as a feedback mechanism for mitochondrial transcription (Ro et al, 2013).

In addition, it has previously been shown that although most mitosRNA localize to the mitochondria some are found within the nucleus, denoting farther gene regulation (Kwon et al, 2014; Liang et al, 2021). We add to the earlier findings by showing that their biogenesis in sperm is increased by a high-sugar diet.

Sperm sRNA profiles, especially alterations in miRNA, have been studied in infertile men (Abu-Halima et al, 2014; Lian et al, 2009; Muñoz et al, 2015; Salas-Huetos et al, 2016; Wang et al, 2011), and they have been suggested as biomarkers of male infertility (Barbu et al, 2021; Kiani et al, 2019; Kotaja, 2014; Salas-Huetos et al, 2020). In addition, sperm tsRNA (Grosso et al, 2021; Nätt and Öst, 2020), miRNA (Xu et al, 2020), and rsRNA (Hua et al, 2019) have recently been shown to correlate with embryo quality.

We have earlier described that a short high-sugar intervention in healthy young men synchronously increases tsRNA and rsRNA coming from the mitochondrial genome and that the increase of mt-tsRNA is positively associated with simultaneous changes in sperm motility (Nätt et al, 2019). Here, by reanalyzing data from Donkin et al (2016), we observed that obesity is associated with less mitosRNA, suggesting a link between mitosRNA and metabolic diseases.

Moreover, the reanalyzing of data from Hong et al (2021) and Hua et al (2019) shows that fertile sperm have a higher level of mitosRNA than infertile sperm, and that sperm-producing embryos of high quality show the same global increase in mitosRNA, respectively. These findings both indicate a regulatory role of mitosRNA and suggest them to be good indicators of sperm quality.

## Materials and Methods

### Fly stock maintenance and sugar diet administration

A standard laboratory strain *W^1118^* was used for proteomics, sRNA sequencing, and certain microscopic experiments in this study. Dj-GFP males were used for ROS experiments to visualize sperm tail in seminal vesicles. The flies were inbred for several generations and maintained on standard cornmeal/molasses media at 26°C. Male flies were isolated within 2 days of eclosure and aged for an additional 2–3 days before switching them to paternal diet intervention food containing 3, 30, or 300 g/L white sugar, for 2 days.

Standard food: Agar 10 g/L, yeast 28 g/L, cornmeal 68 g/L, molasses 68 g/L, Nipagin 1.5 g/L, and propionic acid 5.5 mL/L.

Paternal diet intervention food: Agar 12 g/L, yeast 10 g/L, propionic acid 4,5 mL/L, soy flour 30 g/L, and white sugar as indicated.

### Isolation of seminal vesicles and preparation of protein extracts

Seminal vesicles from 20 males for each diet were isolated in insect medium (Sigma # T3160) using fine forceps and collected in 25 μL ice cold Mili-Q water in a 1.5 mL microfuge tube stored on ice. After dissections of the complete set, tissue was lysed mechanically using a fitted pestle (VWR #431-0094), followed by 2 min (40 oscillation per second) on a bead shaker (Qiagen Tissue lyser) and centrifuged at 1000 *g* for 5 min at 4°C to remove debris; and the supernatant was used for further processing.

Twenty microliters of the supernatant was first alkylated in the presence of 10 m*M* DTT in ammonium bicarbonate (25 m*M*) for 1 h at 56°C. Alkylation of cysteines was performed using 55 m*M* iodoacetamide prepared in ammonium bicarbonate solution (25 m*M*) for 1 h at room temperature in the dark. Following this, proteins were precipitated using ice cold acetone overnight at −20°C. The samples were subsequently centrifuged at 15,000 *g* for 10 min in a cooled rotor, and the supernatant was aspirated out.

The pellet was used for Trypsin digestion (0.005 μg/μL; Pierce #90057) at 37°C overnight. The next morning, after a further boost of trypsin (0.0025 μg/μL) for 3 h at 37°C, the digested peptides were vacuum-dried, and they were stored at −2°C. The peptides were resuspended in 12 μL 0.1% formic acid and used in duplicate (5 μL) for mass-spectrometry analysis.

### Mass spectrometry analysis

The peptides were introduced into an LTQ Orbitrap (Thermo Fisher Scientific, San Jose, CA) mass spectrometer, and all MS/MS samples were analyzed using Sequest (Thermo Fisher Scientific; version IseNode in Proteome Discoverer 1.4.0.288). Sequest was set up to search Fly uniprot 7227.fasta assuming the digestion enzyme trypsin, with the following parameter settings: 1 miscleavages, variable methionine oxidation and phosphorylation on serine and threonine, carboxymethyl cysteine as fixed modification, with a fragment ion mass tolerance of 0.50 Da and a parent ion tolerance of 6.0 PPM. Results were merged using Scaffold (Proteome Software) version 3.00.04.

### Criteria for protein identification

Scaffold (version Scaffold_4.10.0; Proteome Software, Inc., Portland, OR) was used to validate MS/MS-based peptide and protein identifications. Peptide identifications were accepted if they could be established at greater than 95.0% probability by the Scaffold Local FDR algorithm. Protein identifications were accepted if they could be established at greater than 99.0% probability and contained at least two identified peptides.

Protein probabilities were assigned by the Protein Prophet algorithm (Nesvizhskii et al, 2003). Proteins that contained similar peptides and could not be differentiated based on MS/MS analysis alone were grouped to satisfy the principles of parsimony. Proteins sharing significant peptide evidence were grouped into clusters.

### Protein classification

Flybase IDs of the entire list (542 proteins) were analyzed in FlyMine. The tool for pathway enrichment was used, with normalization to gene length, and Benjamini-Hochberg correction factor of maximum *p* value 0.05. Most proteins were not assigned to any pathways. In such cases, information from Uniprot and FlyBase was used to assign a broad category for the protein. Most metabolic proteins were assigned by FlyMine.

Many proteins were involved in more than one pathway, as shown in Supplementary Table S1. Certain proteins, for example Gld and glob1, although known to be involved in redox homeostasis, were not annotated by FlyMine. In such cases, they were assigned to a class manually. The human orthologues are from FlyBase/DIOPT.

### Bioinformatic analysis (proteomics)

The peptide counts for each protein across the diets tested were compiled into a excel file, with individual proteins represented in rows and each sample per diet in columns. Normalization to the mean of sum of all protein counts was performed. Statistical analyses were performed in R ver. 3.6.3. Significant changes were analyzed with a linear model fit in limma ver 3.42.2 (Ritchie et al, 2015) and edgeR ver. 3.28.1 (McCarthy et al, 2012) and adjusted with Benjamini Hochberg for adjusted *p*-values. Thirty g/L was used as an intercept in design. Heatmaps were generated using heatmapper (Babicki et al, 2016) hierarchical clustering based on Pearson complete distance measure. All other statistics were done with GraphPad prism.

### GO term analyses

The GO terms for biological processes were assigned using PantherDB (Mi et al, 2021) with the statistical enrichment tool. Gene names from each cluster (Fig. 1D) were separately analyzed, and the list was sorted based on fold enrichment with reference to the entire genome of *Drosophila*. The fold enrichment was converted to Log_2_ values and plotted with GO terms as a bar graph using GraphPad prism (8.3.0).

### Measurement of ROS by microscopy

After paternal diet intervention for 2 days, seminal vesicles were dissected (from Dj-GFP or W^1118^) in insect medium (Sigma; # T3160) (for CellROX) or 1 × PBS (H_2_DCFDA), and they were subsequently incubated in CellROX orange dye (Invitrogen; # C10443) for 30 min at 37°C (5 μ*M* final concentration in insect medium) or H_2_DCFDA (Invitrogen #D399) at room temperature for 5 min (40 μ*M* final concentration). After this, the tissue was rinsed extensively with 1 × PBS; mounted onto glass slides with halocarbon oil (HC700; Sigma) that served as the mounting medium; and covered with glass coverslips.

Coverslips were sealed with clear nail polish, and slides were imaged using an inverted confocal microscope (LSM800; Zeiss) using absorption/emission maxima of ∼545/565 nm (for CellROX) or ∼488/517 nm (for H_2_DCFDA and GFP). The timing of start of dietary regimen, and that of imaging for ROS was kept constant across all experiments. All images were quantified in Fiji (Schindelin et al, 2012). The steps followed to process the images in Fiji are depicted next. The mean gray values are plotted in graphs.

>>Fiji
>>image import. Czi
>>process>subtract background>rolling ball radius 100 pixels
>>Image>adjust>threshold-set threshold min and max: 30to 255
>>Choose ROI
>>Measure: Integrated density and mean gray values

### Antioxidant supplementation to counteract ROS

N acetyl cysteine (Sigma; # A7250) solubilized in water was used in food containing 30- or 300 g/L sugar at a final concentration of 1 mg/mL, and the intervention was carried out for 2 days at 26°C. After this, testes were dissected, and seminal vesicles were imaged for ROS production using CellROX orange dye as described earlier. Simultaneously, the sperm from 15 flies from each condition was isolated and used for sRNA sequencing as described later.

### RNAi crosses

RNAi was initiated in the male germline by crossing males expressing UAS-RNAi (SOD1: BDSC#29389; eIF4A: BDSC#33970) or UAS-GFP RNAi (BDSC#35786) with virgin Nanos Gal4; UAS Dicer 2 (BDSC#25751) on standard food. Eclosed F1 males were subjected to dietary intervention and ROS measurements using CellROX orange dye as described earlier.

### Imaging for redox analysis

Flies expressing roGFP2-orp1 in the mitochondria (BDSC# 67672) were subjected to dietary intervention, as previously detailed. Flies were dissected in 1 × PBS, and testes were mounted on halocarbon oil and sealed with coverslips. Imaging was performed immediately using confocal microscopy (upright LSM 700; Zeiss). The probe fluorescence was excited at 405 and 488 nm, sequentially and line by line with emission wavelengths ranging between 518 and 580 nm. To determine the dynamic range (DR), testes from the 30 g/L sugar diet condition were either fully oxidized (using 10% H_2_O_2_) or fully reduced (1 m*M* DTT) and immediately imaged with the settings described earlier.

### Quantification of redox changes

Fiji was used to quantify each channel (405 and 488 nm) separately, with background subtraction and thresholding as described earlier. To calculate the fluorescent intensity ratios, the 405 nm value was divided by 488 nm. All ratios were computed in Excel. The DR, which reflects the maximal achievable redox changes in our model, was calculated by dividing the 405/488 ratio of the fully oxidized H_2_O_2_ sample with the same ratio of the fully reduced DTT sample.

### Staining of testes with MitoTracker Red CMXRos

Testes of 1- to 3-day-old males were dissected in PBS and stained in MitoTracker Red CMXRos in PBS (Molecular Probes, Eugene, OR) for 15 min at room temperature (final concentration 1 μ*M*). Tissues were rinsed in 1 × PBS and fixed in 4% paraformaldehyde solution for 10 min at room temperature. After fixation, the tissues were extensively rinsed in 1 × PBS triton (0.2%), and they were mounted on glass slides using halocarbon oil. Coverslips were sealed, and imaging was performed in an inverted LSM 800 confocal microscope with Texas red filter settings (ex/em: 579/599 nm). Quantification was done after background subtraction and thresholding in Fiji, as described earlier.

### ATP measurements

Total ATP was measured in pure sperm from eight males per sample using the luminescence-based ATPlite kit (Perkin Elmer; #6016943) as per the manufacturer's instructions. A fraction of the lysate was used simultaneously to determine total protein content using the BCA method (Pierce; #23225). Total ATP (pmol) was normalized to total protein (μg), and the resulting values were graphed.

### Isolation of sperm for small RNA sequencing

Sperm was isolated in TC-100 Insect Medium (Sigma; #T3160) essentially as described (Öst et al, 2014). From each diet, sperm from 15 flies were dissected and pooled in 1:10 dilution of RNAse inhibitor (Recombinant ribonuclease inhibitor 5000 U; Cat. 2313A Takara); samples were flash-frozen on dry ice and later stored at −80°C. For sperm collection, five samples of each diet were prepared.

In the repeated experiment of three sugar diets for verification of results, six samples per diet were prepared, in total 18 samples.

### Small RNA library preparation and sequencing

The RNA extraction was done using miRNeasy Micro kit (Qiagen; 217084) according to the manufacturer's instructions. Before homogenization, cold steel beads (0.15 g; SSB02-RNA NextAdvance, Troy, NY) were added to frozen samples followed by the addition of 500 ul of prechilled Qiazol (Qiagen). Samples were processed in a Tissue Lyser LT (Qiagen) for 2 + 2 min at 40 oscillations/s.

The RNA quality was studied with BioAnalyzer RNA analysis (5067-1511; Agilent, RNA 6000 nano kit). Small RNA libraries were produced with NEBNext Multiplex SmallRNA Library Prep Kit for Illumina (E7560S, E7580; New England Biolabs), with the customization of a dilution of primers 1:2. The 3´adaptor ligation reaction was carried out at 4°C overnight. To minimize the amount of 2S rRNA, a blocking oligo (5´-TAC AAC CCT CAA CCA TAT GTA GTC CAA GCA-SpcC3 3´) was added to the samples at the 5´ adaptor ligation step (Wickersheim and Blumenstiel, 2013).

Libraries were amplified for 16 cycles and cleaned using Agencourt AMPure XP (Beckman Coulter, Brea, CA). Size selection on amplified libraries was done using TBE gel (EC6265BOX; Invitrogen) 130–165 nt length. The extraction of cDNA from gel was performed with Gel Breaker Tubes (3388-100, IST Engineering) by incubation with buffer included in the NEBNext kit and incubated on a shaker for 1 h at 37°C, flash frozen for 15 min, and again incubated on a shaker. Gel debris was removed by Spin-X 0.45 μm centrifuge tubes (Corning, Inc., Corning, NY). Precipitation was done using GlycoBlue (Invitrogen), 0.1 times the volume of Acetate 3M (pH5.5), and three times the volume of 100% ethanol in −70°C overnight.

Quality of cDNA libraries was studied with BioAnalyzer DNA analysis (5067-1504; Agilent, Agilent High Sensitivity DNA kit, 5067-4626). Final DNA concentration was determined with Quantus Fluorometer (E6150; Promega, Madison, WI) using QuantiFluor ONE ds DNA system. Libraries were pooled and sequenced on NextSeq 500 with NextSeq 500/550 High Output kit version 2.5, 75 cycles (Illumina, San Diego, CA). All libraries passed Illumina's default quality control.

### Bioinformatic analyses (sRNA)

Data analysis was performed with Seqpac ver. 0.99.0 (Skog et al, 2021). Adaptor trimming, quality control, and mapping were all performed in Seqpac with make_counts and make_reanno workflow with evidence that an individual sequence should have at least 1 count in 2 separate samples. Trimming was performed on the adaptor sequence of the used NebNext library (AGATCGGAAGAGCACACGTCTGAACTCCAGTCA). Only reads with an adaptor sequence present before trimming were studied. Averaged over all 20 samples, 1.9 × 10^6^ unique reads passed filtering and with a mean of 1.5 × 10^7^ reads per sample.

Genomic mapping was performed toward *Drosophila* reference dm6 downloaded from UCSC. Biotype mapping was performed to Ensembl ncRNA BDGP6.32, piRNA piRBase *D. Melanogaster* 2.0 (Wang et al, 2019; Yuan et al, 2016; Zhang et al, 2015; Zhang et al, 2014), miRBase 21 (Griffiths-Jones, 2004; Griffiths-Jones et al, 2008; Griffiths-Jones et al, 2006; Kozomara and Griffiths-Jones, 2014; Kozomara and Griffiths-Jones, 2011; Kozomara et al, 2019), protein coding from Ensembl BDGP6.32 (Howe et al, 2021), and tRNA from GtRNAdb (Chan and Lowe, 2016; Chan and Lowe, 2009) Drosophila BDGP dm6.

Human mapping was performed against the human genome GRCh38.p13 (GCA_000001405.28) from Ensembl (Howe et al, 2021). Biotype mapping was performed to Ensembl ncRNA, piRNA piRBase v 2.0 (Wang et al, 2019; Yuan et al, 2016; Zhang et al, 2015; Zhang et al, 2014), miRBase 21 (Griffiths-Jones, 2004; Griffiths-Jones et al, 2008; Griffiths-Jones et al, 2006; Kozomara and Griffiths-Jones, 2014; Kozomara and Griffiths-Jones, 2011; Kozomara et al, 2019), and protein coding from NCBI RefSeq proteins and tRNA from GtRNAdb (Chan and Lowe, 2016; Chan and Lowe, 2009). Biotypes were hierarchically determined in the order rRNA, mitochondrial tRNA, tRNA, miRNA, lncRNA, piRNA, and protein coding. Bowtie indexes were created with Rbowtie ver 1.32.0.

Data were first filtered with function PAC_filter on a size of 18–50 nt length, 10 counts in 60% of samples, and a perfect (no mismatch) match with reference genome. Additional filtering was performed after CPM calculations to remove reads without a presence of minimum 20 counts per million in 25% of samples. Computation of counts per million and Log_2_-fold change; and plots for Figures 3B, C,E, F; 4B, D, and 5B & D were carried out with Seqpac.

Other figures presented were created with ggplot2 ver 3.3.3 and pheatmap ver 1.0.12. Unless stated otherwise, data used in figures are CPM for individual sequences. In Figure 3E, Log_2_-fold change is calculated on a feature base rather than a sequence base, where all sequences mapping to a certain miRNA are classified together.

When comparing two separate sequencing runs for verification, quality control, annotation, and filtering were performed as previously described.

Analysis of tsRNA and their cleavage sites was performed with the PAC_mapper and PAC_trna analytic workflow in Seqpac. Here, ss-files constructed with tRNAscan-SE for the Drosophila nuclear and mitochondrial tRNA were used (Lowe and Chan, 2016). We defined five tsRNA subtypes: 5′-half, 5′-tsRNA, i-tsRNA, 3′-tsRNA, and 3′-half, where a 5′-half starts in the 5′ end of the mature tRNA and ends in the anticodon loop. Further, 5′-tsRNA also starts in the 5′ end but ends before the anticodon loop.

The opposite relationship is true for 3′-halves and 3′-tsRNA, whereas i′-tsRNA are fragments without connection to neither 5′ nor 3′ end. Fragments from tRNA are here reported to the isodecoder and isoacceptor that they originate from. Multimapping information is found in Supplementary Table S3.

Repeated-measure correlation analysis was performed with R package rmcorr version 0.4.5, with sample identity as a participant.

For all source codes used, see https://github.com/signeskog/Ramesh-2022.

### Statistical analyses (sRNA)

Statistical analyses on sRNA data were performed with a Negative Binomial Generalized Linear Model, since the data is count based. The sRNA sequences are in some cases impossible to map to one unique place in the genome, due to their short size and the repetitive nature of some transcription sites. As of now, there is no perfect method to add this uncertainty into a statistical model and we have not accounted for the ambiguity stemming from the risk of multimapping. Since we cannot guarantee that each sequence stems from one original place, we did not perform multiple testing.

In the case of miRNAs, we did not take miRNA isoforms into account, but rather combined sequences originating from the same miRNA. This made it so our model studied the difference on an miRNA-to-miRNA basis rather than a sequence-to-sequence basis. The model was performed with function glm.nb from the R package MASS version 7.3–51.4. Clustering of tsRNA (Fig. 4C) was performed with pheatmap v 1.0.12, where clustering was performed on rows with *k* = 4 and with Euclidean distance. Standard errors of the mean for Log_2_-fold changes were calculated on the SEM of each individual of 30 g/L (*n* = 5) fold change against 300 g/L for each sequence.

As human sperm parameters were collected at three timepoints per individual in Nätt et al (2019), analyses of correlation of mitochondrial sRNA and such parameters were performed with a repeated-measure correlation.

### Data and code availability

Raw sRNA-seq performed in sperm have been deposited at Sequence Read Archive with the accession number PRJNA770968. Proteomics data generated in this study are deposited to ProteomeXchange consortium *via* Pride PXD029265. All codes for this project are available on GitHub at https://github.com/signeskog/Ramesh-2022. An electronic laboratory notebook was not used.

Human data used in this study can be found at the Gene Expression Omnibus (GEO) GSE74426 for Donkin et al (2016), GSE172486 for Hong et al (2021), and GSE110190 for Hua et al (2019). Curated data of human sperm of Nätt et al (2019) are available in the original publication.

## Authors' Contribution

Conceptualization: A.Ö. and R.R.; Methodology: R.R., S.S., U.K., D.N., L.Ö., and A.Ö. Data curation: S.S. Formal analysis, R.R., S.S., D.N., and A.Ö., Resources: A.Ö., Software: S.S., D.N., Visualization: R.R., S.S., D.N., and A.Ö., Writing – original draft: R.R., S.S., and A.Ö., Writing – review & editing: R.R., S.S., U.K., D.N., L.Ö., and A.Ö.

Funding acquisition: A.Ö. Supervision

All authors have approved the final version of this article.

## Supplementary Material

Supplemental data

Supplemental data

Supplemental data

Supplemental data

Supplemental data

Supplemental data

Supplemental data

Supplemental data

Supplemental data

Supplemental data

Supplemental data

Supplemental data

Supplemental data

## Abbreviations Used

DR: dynamic range
ETC: electron transport chain
IGMR: intergenerational metabolic responses
NAC: N-acetyl cysteine
RDI: recommended daily intake
ROS: reactive oxygen species
TCA: tricarboxylic acid cycle
ΔΨm: mitochondrial potential
