# Supplementary material for: Dietary Sugar Shifts Mitochondrial Metabolism and Small RNA Biogenesis in Sperm
Source: Antioxid Redox Signal. 2023 May 25;38(16):1167–83. doi: 10.1089/ars.2022.0049 (PMC10249743; doi:10.1089/ars.2022.0049)
Supplement: Supplemental data [file Suppl_FigS1.docx]

**Supplementary Figure 1**: Proteomic composition of Drosophila sperm

**A** The expression of the top 9 abundant sperm proteins is not affected by dietary sugar as shown in the bar graph. Mean Log2 values are plotted in the bar graph. **B** Schematics of RNAi crossing scheme. RNAi was induced in the germline by crossing virgin female Nanos-Gal4; UAS-Dicer2 flies with males carrying the UAS-RNAi construct. The F1 adults were screened for testes phenotype and subjected to ROS measurements in seminal vesicles using ROS indicators. **C** Light-microscopy image of testes phenotype with and without eIF4A in the germline. DIC images of Nanos Gal4; Dicer2 heterozygous testes shows normal testes (T), accessory glands (AG) and seminal vesicles with sperm (SV). Nanos-Gal4; Dicer2> eIF4A RNAi (eIF4Ai) shows shorter T and lack SV. Scale bar: 700 µm . DIC= differential interference contrast.
