# Supplementary material for: Dietary Sugar Shifts Mitochondrial Metabolism and Small RNA Biogenesis in Sperm
Source: Antioxid Redox Signal. 2023 May 25;38(16):1167–83. doi: 10.1089/ars.2022.0049 (PMC10249743; doi:10.1089/ars.2022.0049)
Supplement: Supplemental data [file Suppl_FigS2.docx]

**Supplementary Figure 2**: Mitochondrial redox is altered by diet

**A** The mito-roGFP2-Orp1 is expressed and visualised in seminal vesicles (top panel) and on sperm (bottom panel). The sequential excitation wavelengths are 405- and 488 nm, and emission was captured at 518-580 nm.  **B** Establishment of dynamic range for measurement of redox changes using the mito-roGFP2-Orp1 sensor. H_2_O_2_ and DTT represent fully oxidised and fully reduced conditions, respectively. Bar graphs represent quantified ratio of fluorescence emission from sequential excitation at 405-and 488 nm. Reduced condition was arbitrarily set at 0.2.  DR= dynamic range. **C** Live imaging of roGFP2-Orp1 in sperm/seminal vesicles after dietary intervention with 30- or 300 g/L sugar. **D** MitoTracker^TM^ Red-CMXRos staining reveals no apparent change in mitochondrial potential (ΔΨm) in sperm/ seminal vesicle.  Flies were fed 3-,30- or 300 g/L sugar diet for two days, seminal vesicles were dissected, stained with the dye, and subjected to paraformaldehyde fixation. Fluorescence microscopy images showing cellular distribution of mitochondria-specific dye MitoTracker^TM^ Red-CMXRos (red) in the sperm within the seminal vesicle. Representative images from the three tested diets are presented **E**: ATP production in sperm mitochondria is not altered by diet. Purified sperm from male flies fed with either 3-, 30- or 300 g/L sugar diets were used to measure ATP. Each coloured circle represents one sample containing sperm from 8 males**.**
