# Supplementary material for: Dietary Sugar Shifts Mitochondrial Metabolism and Small RNA Biogenesis in Sperm
Source: Antioxid Redox Signal. 2023 May 25;38(16):1167–83. doi: 10.1089/ars.2022.0049 (PMC10249743; doi:10.1089/ars.2022.0049)
Supplement: Supplemental data [file Suppl_FigS3.docx]

**Supplementary Figure 3:** **Changes in miRNA after dietary intervention**

**A**: DNA staining was used to determine purity of sperm used for sRNA seq. Seminal vesicles containing mature sperm (top panel) were dissected out carefully using fine forceps and sperm were pulled out for sRNA-seq (lower-left panel). Sperm- and somatic nuclei reveal distinct nuclear morphologies (indicated by pink and green arrows respectively, in top panel), allowing for the determination of sperm purity. As shown in the lower panel, no somatic nuclei are present in the dissected fraction used for sRNA seq. **B**: Changes in miRNA after dietary intervention. All miRNA identified in the study in fly sperm are presented under the fly pictogram. The corresponding human orthologues, where present, are indicated under the human pictogram. Bar graphs in descending order represent abundance (Log_10_CPM) of each miRNA in both diets of 30 and 300 g/L, with individual samples indicated with closed circles and error bars representing ± SEM. Each miRNA represents a mean of all transcripts annotated to that miRNA. Fold changes between 30- vs 300 g/L are indicated as closed circles to the right.  Statistically significant miRNAs (*p<0.05) were calculated by Generalized Linear Negative Binomial model. Results are mean ± SEM.
