# Supplementary material for: Dietary Sugar Shifts Mitochondrial Metabolism and Small RNA Biogenesis in Sperm
Source: Antioxid Redox Signal. 2023 May 25;38(16):1167–83. doi: 10.1089/ars.2022.0049 (PMC10249743; doi:10.1089/ars.2022.0049)

**Supplementary Figure 4:** Dietary shifts in miRNA of 3, 30 and 300 g/L sugar diet in Drosophila sperm**. A**: Heatmap of mean CPM of mature miRNAs per dietary condition. Each row represents one miRNA and each column represents one diet. **B**: Expression levels of each individual sample over miRNA. Y-axis represents mean CPM and each box contains sequences mapping to each miRNA as stated above the box. Each point represents the mean expression per miRNA of one sample. Boxplots are split up dependent of dietary regime of flies, as stated in the legend, with increase of sugar levels from left to right.


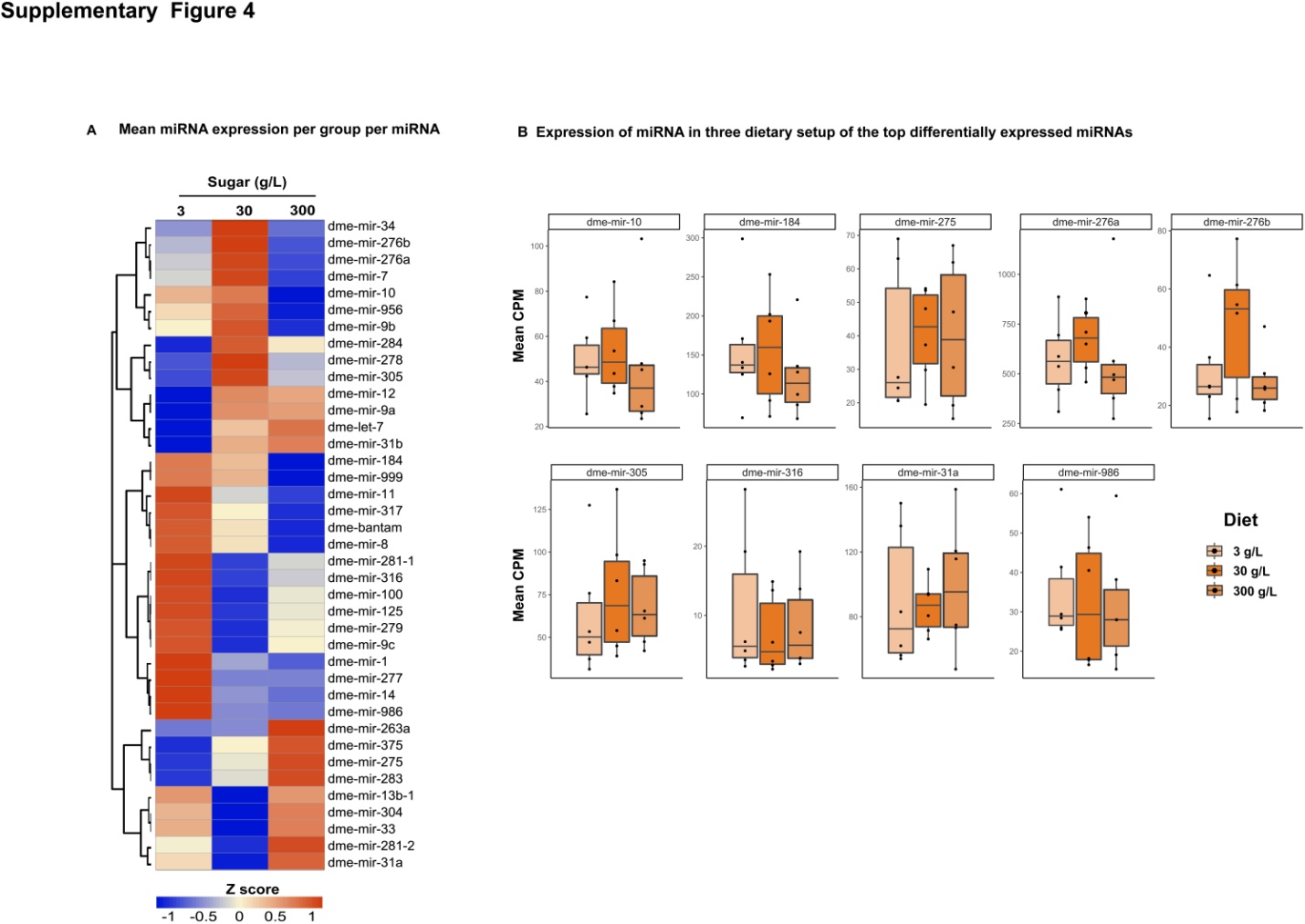

Supplement: Supplemental data [file Suppl_FigS4.docx]
