# Supplementary material for: Dietary Sugar Shifts Mitochondrial Metabolism and Small RNA Biogenesis in Sperm
Source: Antioxid Redox Signal. 2023 May 25;38(16):1167–83. doi: 10.1089/ars.2022.0049 (PMC10249743; doi:10.1089/ars.2022.0049)
Supplement: Supplemental data [file Suppl_FigS5.docx]

**Supplementary Figure 5: Effects of dietary sugar on tsRNA**

**A**: Heatmap of transcripts mapping to tRNA.Data shown here are the z values of mean for all transcripts mapping to mature tRNA, where columns represent mean for one diet and rows represents each tRNA found. **B**: Coverage graphs of mature mitochondrial tRNA reaching a mean above 30 CPM. X axis represents the transcript sequence, and y axis represents the mean CPM.
